# Lack of Evidence of COVID-19 Being a Risk Factor of Alopecia Areata: Results of a National Cohort Study in South Korea

**DOI:** 10.3389/fmed.2021.758069

**Published:** 2021-10-13

**Authors:** Jeehyun Kim, Kwan Hong, Raquel Elizabeth Gómez Gómez, Soojin Kim, Byung Chul Chun

**Affiliations:** ^1^Graduate School of Public Health, Korea University, Seoul, South Korea; ^2^Department of Preventive Medicine, Korea University College of Medicine, Seoul, South Korea; ^3^Transdisciplinary Major in Learning Health Systems, Department of Healthcare Sciences, Graduate School, Korea University, Seoul, South Korea

**Keywords:** COVID-19, SARS-CoV-2, complications, alopecia areata, cohort study

## Abstract

**Background:** Concerns about alopecia areata (AA) in coronavirus disease 2019 (COVID-19) patients have emerged among dermatologists. However, most of the extant kinds of literature have limited implications by relying on cross-sectional studies with restricted study subjects without the control group.

**Objective:** Our study aims to investigate the risk of developing AA among COVID-19 patients in South Korea using national representative data.

**Methods:** We used the National Health Insurance Service COVID-19 cohort database, comprising COVID-19 patients and the control group, all of whom were diagnosed from January 1, 2020, to June 4, 2020. Patients were defined as individuals who were confirmed as COVID-19 positive, regardless of disease severity. Controls were defined as those who were confirmed as COVID-19 negatives. People with a history of AA during the period 2015–2019 were excluded. The primary endpoint was a new diagnosis of AA (ICD-10-Code: L63). The adjusted incidence rate ratio (IRR) of developing AA was estimated using a log-link Poisson regression model based on incidence density. The model adjusted for (1) age and sex and (2) demographic variables (age, sex, place of residence, and income level).

**Results:** A total of 226,737 individuals (7,958 [3.5%] cases and 218,779 [96.5%] controls) were included in the final analysis. The ratio of newly diagnosed AA was 18/7,958 (0.2%) in cases and 195/218,779 (0.1%) in controls. IRRs of COVID-19 patients having newly diagnosed AA compared to controls were 0.78 (95% CI: 0.48–1.27) when age and sex were adjusted for and 0.60 (95% CI: 0.35–1.03) when all demographic variables were adjusted for.

**Conclusion:** Diagnosis of COVID-19 was not significantly associated with the development of AA even after appropriately adjusting for covariates.

## Introduction

There have been approximately 170 million coronavirus disease 2019 (COVID-19) pandemic survivors worldwide by July 9, 2021 (1). As a result, concerns about alopecia areata (AA), an autoimmune disease characterized by nonscarring hair loss (2), in COVID-19 patients have emerged among dermatologists. COVID-19 is suggested to be an additional risk factor of AA, in the way that COVID-19 itself affects autoimmune pathogenesis of AA (3–5) or that psychological stress caused by restriction policy against COVID-19 burdens AA (3–8). However, there is a study that mild to moderate COVID-19 is not associated with worsening AA (9).

This inconsistency might result from limitations of extant literature: relying on cross-sectional studies with restricted study subjects without the control group (4, 6, 8–10), based on a small sample size that is not national representative (3–9). Furthermore, the study based on South Korea lacks even though South Korea having about 152,000 survivors (1). Therefore, our study aims to investigate the risk of developing AA among COVID-19 patients in South Korea using adequate control based on nationally representative data.

## Materials and Methods

### Study Design and Participants

We used the National Health Insurance Service (NHIS) COVID-19 cohort database, comprising COVID-19 patients and the control group among national health insurance subscribers in South Korea, all of whom were diagnosed from January 1, 2020, to June 4, 2020.

The NHIS COVID-19 cohort database was developed in cooperation between NHIS and Korea Centers for Disease Control and Prevention (KCDC) for research and opened for about 5 days per research team. Information (official announcement date of COVID-19 diagnosis, treatment outcomes, and demographic information) of COVID-19 patients and controls, all of whom were diagnosed from January 1, 2020, to June 4, 2020, were provided from the KCDC to the NHIS COVID-19 cohort database. Patients were defined as individuals who were confirmed as COVID-19 positive using SARS-CoV-2 real-time reverse transcription PCR test or virus isolation (11), regardless of disease severity. Controls were defined as those who were confirmed as COVID-19 negatives. NHIS provided information of demographic, medical history, prescription, results of medical checkups, and death of COVID-19 patients and controls. The database excluded foreign COVID-19 patients.

### Statistical Analysis

We excluded people with a history of AA during the period 2015–2019 from the database to investigate newly developed AA after the COVID-19 diagnosis. The start date of the follow-up for cases was the diagnosis date. However, the diagnosis date in the data could be different from the real-diagnosis date because the official announcement date of diagnosis was used as the diagnosis date because of the privacy protection. Furthermore, the last date of COVID-19 diagnosis of the case group, June 4, 2020, was used as the start date of the follow-up for controls, as the COVID-19 diagnosis date of controls was not provided to protect privacy. The primary endpoint was a new diagnosis of AA (ICD-10-Code: L63).

**Figure 1:**
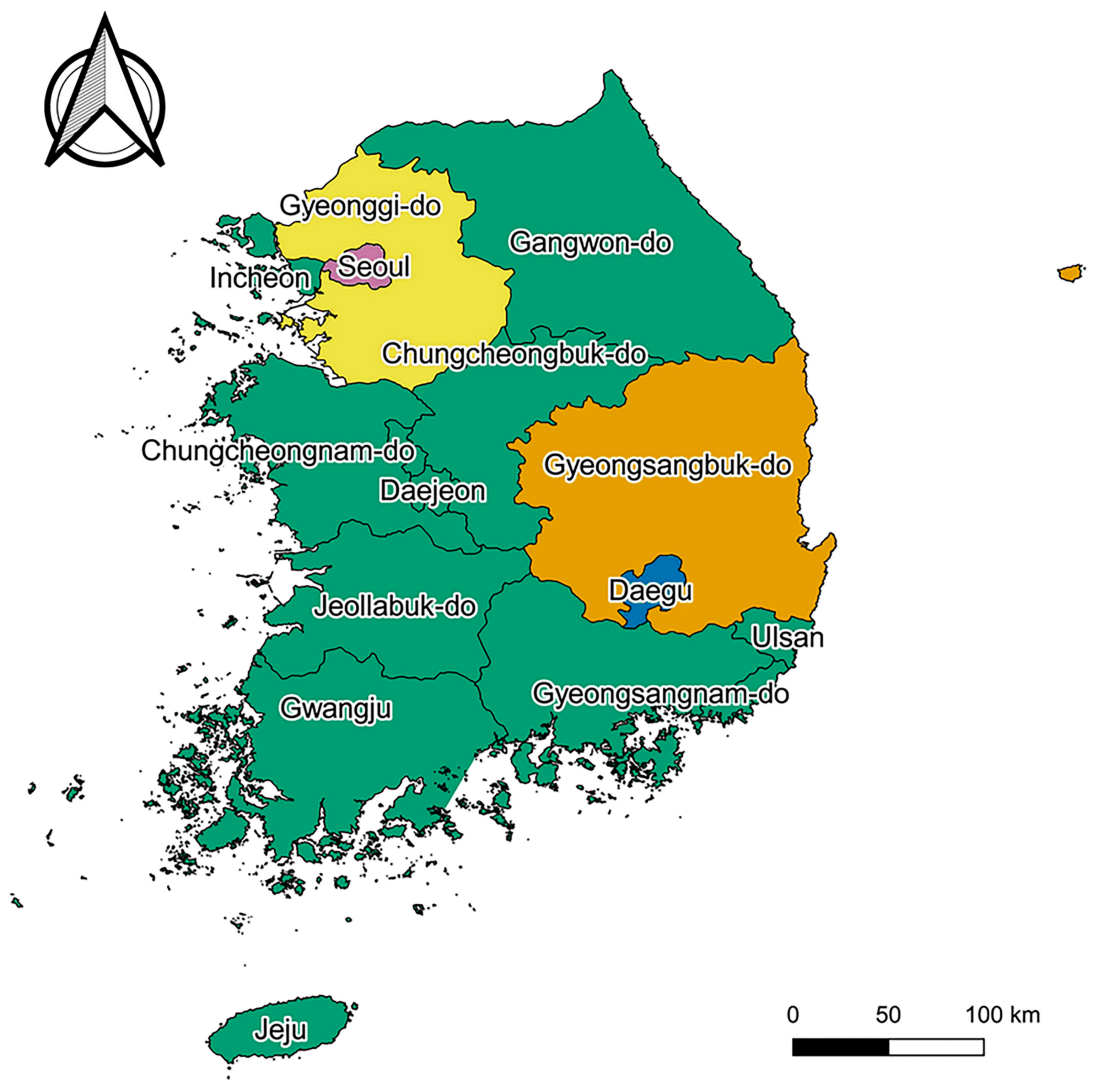
Map of South Korea. Pink, yellow, blue, orange, and green regions represent “Seoul,” “Gyeonggi-do,” “Daegu,” “Gyeongsangbuk-do,” and “others,” respectively. Map created by Kim et al. (2021), using QGIS version 3.10.13 (QGIS Development Team, http://qgis.osgeo.org). The Shapefile used in this figure was obtained from “South Korea level 1” of GADM data version 3.6 (https://gadm.org/download_country_v3.html), the data to create maps for academic publishing is freely available (License suggested: https://gadm.org/license.html).

Demographic variables, including age (12, 13), sex (12, 13), place of residence (13, 14), and income level (13, 15), were considered as the potential confounders in the association between COVID-19 diagnosis and AA development. Age in 2020 was categorized into nine groups (0–9; 10–19; 20–29; 30–39; 40–49; 50–59; 60–69; 70–79; and ≥ 80 years). Place of residence in 2020 was categorized into five groups: Seoul (11), Gyeonggi-do (41), Daegu (27), Gyeongsangbuk-do (47), and others (99) (Figure 1). Income level in 2020 was used as a continuous variable, having a value from 0 (the lowest) to 20 (the highest). In addition, health behaviors [drinking alcohol frequency (16–18), smoking history (16, 17, 19)], comorbidities [diabetes mellitus (20, 21), heart disease (22), hypertension (20, 23), and dyslipidemia (20, 24)], anthropometric values [body mass index (19, 20, 25), glomerular filtration rate (26), gamma-glutamyl transferase level (27), hemoglobin level (28), and height (29)], and covariates (family history of heart disease) being considered as the secondary potential confounders, the variables were only adjusted for the sensitivity analysis with Cox proportional regression.

Descriptive statistics, independent sample *t*-tests for continuous variables, and chi-squared tests for categorical variables were conducted to analyze the demographics of COVID-19 patients and the negatives. Adjusted incidence rate ratio (IRR) of developing AA was estimated using log-link Poisson regression model based on incidence density of case and control group. The models were adjusted for (1) age and sex and (2) demographic variables. For sensitivity analysis, first, Cox proportional regression was performed with adjusting for demographic variables, health behaviors, comorbidities, anthropometric values, and covariates. In addition, different start date of controls' follow-up (January 1, 2020; the first date of the first year when COVID-19 patient was detected in South Korea) was used for two log-link Poisson regression models, which adjusted for (1) age and sex and (2) demographic variables, and one Cox proportional regression model, which adjusted for demographic variables, health behaviors, comorbidities, anthropometric values, and covariates, as sensitivity analysis.

All statistical analysis was performed in SAS Enterprise Guide version 7.13 (SAS Institute Inc., Cary, NC, USA) and statistical significance was set at *p* < 0.05. This study was exempted from ethical approval by the Institutional Review Board (IRB) of Korea University (IRB exemption number: KUIRB-2019-0036-02).

## Results

**Figure 2:**
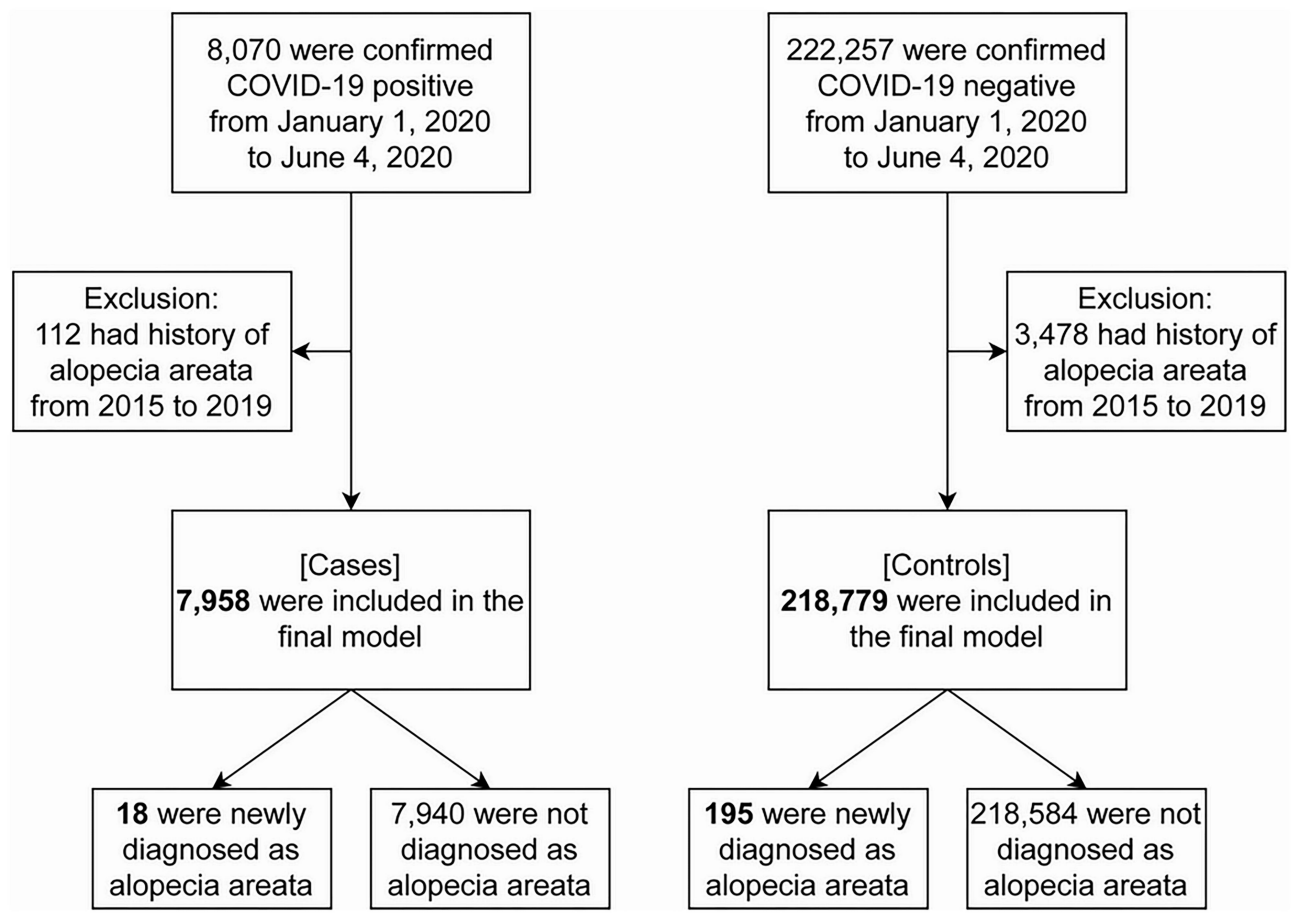
Flowchart of study subject selection. Alopecia areata was diagnosed with L63 in ICD-10-Code. COVID-19, coronavirus disease 2019.

While the NHIS COVID-19 database contained 8,070 COVID-19 patients and 222,257 controls, 112 patients and 3,478 controls were excluded by having a history of AA from 2015 to 2019. Therefore, a total of 226,737 individuals (7,958 [3.5%] cases and 218,779 [96.5%] controls) were included in the final analysis (Figure 2). Table 1 depicts descriptive statistics of cases and controls. The ratio of newly diagnosed AA was 18/7,958 (0.2%) in cases and 195/218,779 (0.1%) in controls. There were more women than men, both in test positives and negatives at 59.9 and 52.3%, respectively. The largest test positive population was those in the age group 20–29 years (25.5%). The test negatives had the largest population in the age group 30–39 years (17.1%). In this bivariate analysis, newly diagnosed AA and the demographic features (age, sex, place of residence, and income level) are significantly different between the two groups (*p* < 0.001).

**Table 1:** Demographics of study subjects.

	**COVID-19 patients**		**COVID-19 negatives**		*p*-value^*^
	**(*****N*** **=** **8,070)**		**(*****N*** **=** **222,257)**		
Newly diagnosed alopecia areata					<0.001
(without alopecia areata history), *n*/total (%)					
Yes	18/7,958	(0.2)	195/218,779	(0.1)	
No	7,940/7,958	(99.8)	218,584/218,779	(99.9)	
**Demographic variables**
Age (years of age), *n* (%)					<0.001
0–9	81	(1.0)	10,081	(4.5)	
10–19	276	(3.4)	7,211	(3.2)	
20–29	2,057	(25.5)	37,506	(16.9)	
30–39	832	(10.3)	37,977	(17.1)	
40–49	1,036	(12.8)	31,361	(14.1)	
50–59	1,567	(19.4)	29,093	(13.1)	
60–69	1,199	(14.9)	25,899	(11.7)	
70–79	617	(7.6)	22,087	(9.9)	
≥80	405	(5.0)	21,042	(9.5)	
Sex, *n* (%)					<0.001
Female	4,834	(59.9)	116,175	(52.3)	
Male	3,236	(40.1)	106,082	(47.7)	
Place of residence, *n* (%)					<0.001
Seoul	544	(6.7)	49,557	(22.3)	
Gyeonggi-do	451	(5.6)	55,662	(25.0)	
Daegu	5,264	(65.2)	21,997	(9.9)	
Gyeongsangbuk-do	956	(11.8)	13,121	(5.9)	
Others	855	(10.6)	81,920	(36.9)	
Income level, mean (SD)	10.0	(6.8)	11.4	(6.3)	<0.001
**Health behaviors**
Drinking alcohol frequency, mean (SD)	1.2	(2.6)	1.6	(2.7)	<0.001
Smoking history, *n* (%)	626	(14.4)	21,406	(16.6)	<0.001
**Comorbidities**
Diabetes mellitus, *n* (%)	430	(14.4)	13,368	(13.5)	0.157
Heart disease, *n* (%)	112	(3.9)	5,362	(5.6)	<0.001
Hypertension, *n* (%)	877	(27.4)	28,571	(27.3)	0.835
Dyslipidemia, *n* (%)	295	(10.1)	9,361	(9.6)	0.398
**Anthropometric values**
Body mass index (BMI, *kg*/*m*^2^), mean (SD)	24.0	(3.4)	23.9	(3.7)	0.013
Glomerular filtration rate, mean (SD)	90.3	(22.5)	91.7	(28.1)	<0.001
Gamma-glutamyl transferase level (*IU*/*l*), mean (SD)	30.7	(42.7)	38.0	(63.9)	<0.001
Hemoglobin level (*g*/*dl*), mean (SD)	13.7	(1.6)	13.9	(1.7)	<0.001
Height (cm), mean (SD)	161.8	(8.9)	163.6	(9.4)	<0.001
**Covariates**
Family history of heart disease, *n* (%)	244	(8.5)	6,247	(6.5)	<0.001

* *p-values were calculated with independent sample t-tests or chi-squared tests*.

Incidence rate ratios (IRRs) of COVID-19 patients having newly diagnosed AA compared to controls were 0.78 (0.48–1.27) when age and sex were adjusted for and 0.60 (0.35–1.03) when all demographic variables were adjusted for (Table 2). Every result of the sensitivity analysis did not show a significant effect of COVID-19 diagnosis on developing AA, suggesting our results being robust.

**Table 2:** Results of log-link Poisson regression and Cox proportional regression by the different start dates of follow-up of control.

	**Start date of controls' follow-up**	
	**June 4, 2020**	**January 1, 2020**
Adjusted IRR (95 CI)^*^	0.78 (0.48–1.27)	1.13 (0.71–1.81)
Adjusted IRR (95 CI)^†^	0.60 (0.35–1.03)	0.98 (0.60–1.60)
Adjusted HR (95 CI)^‡^	0.47 (0.06–3.50)	1.58 (0.68–3.67)

*IRR, incidence rate ratio; HR, hazard ratio*.

* *Adjusted for age and sex*.

† *Adjusted for age, sex, place of residence, and income level*.

‡ *Adjusted for demographic variables, health behaviors (drinking alcohol frequency and smoking history), comorbidities (diabetes mellitus, heart disease, hypertension, and dyslipidemia), anthropometric values (body mass index, glomerular filtration rate, gamma-glutamyl transferase level, hemoglobin level, and height), and covariates (family history of heart disease)*.

## Discussion

Our results imply that the diagnosis of COVID-19 was not significantly associated with the development of AA even after appropriately adjusting for covariates. Notwithstanding study in Turkey showed an increase in the percentage of patients with AA after the COVID-19 pandemic compared before the pandemic (6) and several case reports suggested the possibility of an association between COVID-19 diagnosis and development of AA (3, 5, 8), our study based on South Korea resulted in that COVID-19 diagnosis not having significant association with new onset of AA. This inconsistency could have arisen because of the study design; our study subject being national representative, while previous studies based on a small sample size that could not perform adequate statistical analysis; our study having an adequate control group to ensure internal validity. Considering the prevalence of AA having racial differences (30), the difference in racial composition in studies might result in inconsistency. Since the database we used excluded foreign COVID-19 patients, the race of the study subjects was relatively homogeneous (31). A South Korea-based previous study that examined the effect of underlying skin disease on COVID-19 susceptibility and mortality also detected no association between skin disease and COVID-19 (32). However, previous studies were mostly based on western society (3–5, 7–9), where the racial composition is different from South Korea. Also, the country where the research is based could derive different results. As timing, category, and stringency of intervention against COVID-19 (33) and COVID-19 pandemic situation (1) vary by country, the level of stress that people get from COVID-19 pandemic would vary by country. Thus, the increase in AA incidence in the COVID-19 pandemic might vary by country, considering emotional stress burden being a risk factor of developing AA (6).

Our study has some limitations. First, the official announcement date, not the real diagnosis date, was used as the COVID-19 diagnosis date because of the privacy policy of the data provider. However, because the official announcement date is usually after the real diagnosis date, it is expected to produce a bias toward the null. In addition, the start date of follow-up of controls, June 4, 2020, was arbitrary. However, the results of sensitivity analysis, which used different start dates (January 1, 2020) that could make the results toward null, showed the consistency of the results. At last, the follow-up period could not be long enough to detect the long-term effect of COVID-19 on AA. However, as the extant case reports suspected COVID-19 being risk factor of developing AA in rapid, about 1–3 months after the onset of COVID-19 (5, 7, 8), the follow-up period of our study, a maximum of 4 months, was sufficient for the analysis. Although there is a possibility that the epidemiology of development of AA varies by the COVID-19 severity (9, 34), the association could not be analyzed because the dataset did not provide the information on the COVID-19 severity. Our research that used a well-structured cohort with an adequate control group implies that diagnosis of COVID-19 did not significantly affect AA development even after appropriately adjusting for covariates. A well-structured cohort database with adequate controls that encompass various countries should be established, stably provided to the researcher so that further study can suggest robust and practical evidence for clinical practices.

## Data Availability Statement

Publicly available data were analyzed in this study. The data can be downloaded through proper procedure from here: https://nhiss.nhis.or.kr.

## Ethics Statement

The studies involving human participants were reviewed and approved by Institutional Review Board of Korea University (IRB exemption number: KUIRB-2019-0036-02). Written informed consent for participation was not required for this study in accordance with the national legislation and the institutional requirements.

## Author Contributions

JK, KH, and BC contributed to the conception or design of the work. JK and KH contributed to the acquisition and analysis of data. JK drafted the manuscript. All authors critically revised the manuscript, contributed to the interpretation of the work, and gave final approval and agree to be accountable for all aspects of work ensuring integrity and accuracy.

## Conflict of Interest

The authors declare that the research was conducted in the absence of any commercial or financial relationships that could be construed as a potential conflict of interest.

## Publisher's Note

All claims expressed in this article are solely those of the authors and do not necessarily represent those of their affiliated organizations, or those of the publisher, the editors and the reviewers. Any product that may be evaluated in this article, or claim that may be made by its manufacturer, is not guaranteed or endorsed by the publisher.
